# Evaluation of a reproductive health awareness program for adolescence in urban Tanzania-A quasi-experimental pre-test post-test research

**DOI:** 10.1186/1742-4755-8-21

**Published:** 2011-06-27

**Authors:** Frida Madeni, Shigeko Horiuchi, Mariko Iida

**Affiliations:** 1 St. Luke's College of Nursing, Maternal Infant Nursing and Midwifery, 10-1 Akashi-cho, Chuo-ku, Tokyo 104-0045, Japan

**Keywords:** adolescent, pregnancy, reproductive health, program evaluation, Tanzania

## Abstract

**Background:**

Sub-Saharan Africa is among the countries where 10% of girls become mothers by the age of 16 years old. The United Republic of Tanzania located in Sub-Saharan Africa is one country where teenage pregnancy is a problem facing adolescent girls. Adolescent pregnancy has been identified as one of the reasons for girls dropping out from school. This study's purpose was to evaluate a reproductive health awareness program for the improvement of reproductive health for adolescents in urban Tanzania.

**Methods:**

A quasi-experimental pre-test and post-test research design was conducted to evaluate adolescents' knowledge, attitude, and behavior about reproductive health before and after the program. Data were collected from students aged 11 to 16, at Ilala Municipal, Dar es Salaam, Tanzania. An anonymous 23-item questionnaire provided the data. The program was conducted using a picture drama, reproductive health materials and group discussion.

**Results:**

In total, 313 questionnaires were distributed and 305 (97.4%) were useable for the final analysis. The mean age for girls was 12.5 years and 13.2 years for boys. A large minority of both girls (26.8%) and boys (41.4%) had experienced sex and among the girls who had experienced sex, 51.2% reported that it was by force. The girls' mean score in the knowledge pre-test was 5.9, and 6.8 in post-test, which increased significantly (t = 7.9, p = 0.000). The mean behavior pre-test score was 25.8 and post-test was 26.6, which showed a significant increase (t = 3.0, p = 0.003). The boys' mean score in the knowledge pre-test was 6.4 and 7.0 for the post-test, which increased significantly (t = 4.5, p = 0.000). The mean behavior pre-test score was 25.6 and 26.4 in post-test, which showed a significant increase (t = 2.4, p = 0.019). However, the pre-test and post-test attitude scores showed no statistically significant difference for either girls or boys.

**Conclusions:**

Teenagers have sexual experiences including sexual violence. Both of these phenomena are prevalent among school-going adolescents. The reproductive health program improved the students' knowledge and behavior about sexuality and decision-making after the program for both girls and boys. However, their attitudes about reproductive health were not likely to change based on the educational intervention as designed for this study.

## Background

Adolescent pregnancy is a top concern among public health problems and is a challenging issue because pregnancy at a young age will include high rates of school dropout and poverty [1,2]. A study in South Africa concerned reproductive health knowledge and pregnancy related school dropouts. They reported that young adolescents with high educational aspirations were less likely to become pregnant while they were enrolled in school.

Sub-Saharan Africa is among the countries where 10% of girls become mothers by the age of 16 years old [3]. The United Republic of Tanzania located in Sub-Saharan Africa is one country where teenage pregnancy is a problem facing adolescent girls. Adolescent pregnancy has been identified as one of the reasons for girls dropping out from school. According to the Tanzania Ministry of Education [4] statistics, 28,600 girls left school between 2004 and 2008 because they were pregnant. The primary school students' dropout from school in 2007 due to pregnancy was 5.6%; while in secondary school, girls' dropping out due to pregnancy was 21.9% [5].

Among factors mentioned which contributed to Tanzanian school girls' pregnancy were poverty, rape, early marriage, and distance from school [6]. According to the study of 197 adolescent girls who aborted illegally, most had sexual intercourse with older men and some had sexual intercourse to obtain money or gifts in exchange for sex (called "sugar daddy phenomenon"), which increased their vulnerability to sexually transmitted diseases (STDs) and HIV/AIDS risk [7]. Another study reported about the "sugar daddy phenomenon" as one of the factors influencing sexual abuse in Tanzania [8].

A study on rural adolescents reported that school children in the rural area of the Mtwara region in Tanzania lack credible knowledge about safe sex [9]. Using a sample of 2,749 including girls and boys, a cross-sectional survey was conducted among 'in school' and 'out of school' unmarried adolescents 10 to 19 years old  [10]. They reported that more than 32% of adolescents were sexually active, which indicated the importance of sexual education for girls and boys in the school environment.

Many young people become sexually active at an early age, yet lack fundamentally important knowledge and skills. A study of a group of 15 girls participating in a method for school-based adolescent sexual education was held in Zaria, Nigeria [11]. This project provided correct information about sexual matters for adolescents to make informed choices and equip them with life-long skills concerning reproductive health. Bearinger et al [12] recognized that boys and girls needed equal knowledge concerning reproductive health to reduce risk behaviors and to promote sexual health. However, the number of studies including boys is limited. Spear and Lock who reviewed 22 articles to examine qualitative research on adolescent pregnancy, found that less than half of the studies included male subjects, and fewer males participated in the individual studies compared to females [13].

Studies conducted in South Africa indicate that early reproductive health programs are important for teenagers because young people become sexually active while they are enrolled in school [14]. However, peer education was also reported to support young people in their decision-making during adolescence because friends are the main source of information about sexual practices and peer pressure [15].

Gallant and Maticka-Tydale reviewed 11 school-based HIV/AIDS risk reduction programs for youths in Africa [16]. They concluded that although there are some limitations, school-based HIV/AIDS prevention programs targeting youth can be successful in changing knowledge and attitudes, and in certain conditions, also behavior.

Pregnancy in unmarried adolescents poses serious problems because it comes at a time when the mother is not yet ready for parenting physically, mentally or financially. In addition, becoming pregnant at a young age also increases risks to the mother and child. The first priority is to provide knowledge about reproductive health; the second is to educate this young generation to make appropriate decisions for their daily life. These actions will help to increase educational opportunities for girls and boys and encourage girls to stay in school longer.

Previous studies conducted in Tanzania based on school-going students' reproductive health have focused largely on STDs, motherhood, sexuality, and family planning programs [7,9,10,17,18]. Education about HIV and STDs have had some success, but there is no focus on decision-making by them for future plans, or to have time to share discussions between both girls and boys. This study attempted to focus on decision-making for future plans for adolescents.

### Purpose of the study

This study's purpose was to evaluate a reproductive health awareness program for the improvement of reproductive health for unmarried adolescent girls and boys in urban Tanzania using a questionnaire assessing their knowledge, attitude, and behavior.

## Methods

### Research design

A quasi-experimental pre-test, post-test research design was conducted to evaluate teenagers' knowledge, attitude, and behavior about reproductive health before and after the program.

### Settings and samples

#### Settings

Dar es Salaam, the capital city of Tanzania, is divided into three districts: Kinondoni to the north, Ilala in the center, and Temeke to the south. The Dar es Salaam Region has a population of 2,497,940 [19] and the city has one referral hospital and each district has one district hospital. This study was conducted in three of the schools in the Ilala district.

One of the researchers stayed in Dar es Salaam and collected data from June to September, 2010.

#### Study population

The inclusion criteria were: school girls and boys between the ages of 11 to 16 years old, the reason being that the youngest reported age at which girls become sexually active in Tanzania was 11 years old [20].

#### Sample size

The questionnaire used in this study consisted of 23 items. When conducting statistical analysis, the sample required is five to ten times the number of items, which is 115 to 230. Considering the follow-up rate to be 80%, the approximate sample size needed will be 300.

### Program development

#### Program objectives

The objectives of this reproductive health education program were (1) to teach and provide basic knowledge of the changes that occur in adolescence, and (2) to provide the opportunity for students to think about the decisions they may make in the future.

#### Program name

The program name "For a Better Tomorrow" (Kesho iliyo njeme) means a program that prepares adolescents to meet their future plans in order to help them obtain quality of life for their future.

#### Program contents

This was a 45-minute program, which was conducted using a picture drama and reproductive health materials. First, students took the pre-test. Next, the researcher FM conducted the lecture. Reproductive health materials used included a Maggie apron picture http://joicfp.or.jp/eng/audio_visual/maggie.shtml, and audio visual aids such as a blackboard and posters. Then a discussion session followed to make adolescents aware of puberty, pregnancy, peer pressure, and outcomes of unprotected sex. This also gave the students an opportunity to clarify the study and learn about the ideas and experiences of their peers. After the discussion there was a post-test. This means that the interval between pre-test and post-test was approximately 45 minutes.

The picture material included 14 pieces of drama material measuring 30 cm by 42 cm. The picture drama used two different trees with adolescents at the top of the trees indicating the two different decision-making paths for young adolescents who engaged in sexual activities and their negative consequences. It also explained positive ways to stay healthy and explained the challenge that having sex early can spoil their future plans and shorten their lives [21].

The Maggie apron is an educational kit for reproductive system education, which facilitates demonstration of the male and female reproductive system and related topics, especially to adolescents and young adults.

#### Instruments

The researchers developed a questionnaire that met the study purpose. The items of the questionnaire focused on the student's knowledge, attitude, and behavior about reproductive health matters based on literature review.

The questionnaire was translated to Kiswahili as a language familiar to most Tanzanians. Data was gathered by an anonymous questionnaire.

The knowledge test consisted of 10 items and asked students if the question was true or false: 0) *false *or 1) *true*. Scores ranged from 0 to 10 points. The higher the score the more knowledge they have about reproductive health. Detailed items are shown in the Result section. The attitude test consisted of seven items and asked students if they agreed or disagreed: 1) *strongly disagree *to 5) *strongly agree*. The possible score range was from 7 to 35 points. High scores mean that they can escape from situations that put them in danger of pregnancy or HIV/AIDS. Examples are: "Girls can say no when they don't want to be touched by boys", "Girls accept sex only because they want gifts or money (reverse)". The behavior test consisted of six items and asked students if they agreed or disagreed: 1) *strongly disagree*, to 5) *strongly agree*. Possible scores ranged from 6 to 30 points. A high score means good decision-making for saying no to sexual behavior. Examples are: "A boy can avoid impregnating a girl if he can avoid sex or use condoms", "I want to have sex with my boyfriend/girlfriend before marriage because I love him/her (reverse)".

The instrument was assessed for the content validity by two experienced nurse researchers. The instruments were pilot tested upon 30 students who were similar to the samples but not from the actual samples. The pilot adolescents encountered no problems with the instruments used in this study; just minor corrections were made for some questions. No changes were made to the instruments.

#### Process evaluation

The process evaluation focused on the educational contents and program operation. The contents discussed were: (1) convenient hours for the program; (2) useful things they learned from the program; (3) appropriate material used in the study; and (4) venue. One of the authors (MF) conducted the process evaluation immediately after the program.

### Data analysis

Descriptive data were used to describe the characteristics demographics. The educational effects were compared using the average score in the pre-test and post-test in each group of girls and boys. The three questionnaires were marked and the difference between the average scores was analysed (paired t-test, level of significance 0.05 bilateral. Statistical analysis software: SPSS ver.17 for Windows).

### Ethical consideration

The Ethics Research Committee of St. Luke's College of Nursing and Tanzania National Institute for Medical Research (NIMR) provided clearance for this study. The District Executive Director and District Education Officer in Tanzania provided permission to conduct the study in their district. Informed consent to participate in the study was sought from the respondents with confidentiality assured when conducting the survey. The head teacher and all school teachers provided permission to conduct the survey at their school but were not permitted to join our survey.

## Results

### Demographic characteristics

In total, 313 questionnaires were distributed to students in grade six and seven. Among these, eight were excluded because of insufficient data and 305 (response rate 97.4%) ended up in the final analysis.

The demographics are shown in Table 1. Respondents comprised 153 girls and 152 boys. Girls' ages ranging from 11 to 12 was 49.7%; 13 years old was 39.2%; and age 14 to 16 was 11.1%. The mean age for girls was 12.5 (SD = 0.9). Boys' ages ranging from age 11 to 12 and 13 years were both 31.6% and ages 14 to 16 was 36.8%. The mean age for boys was 13.2 (SD = 1.2). Christians and Muslims were about half for both girls and boys. The distribution of future plans reported was similar for girls and boys; 85% of the girls planned to go to secondary school after completing primary education and 12.4% planned to find a job, while 71.7% of the boys were planning to go to secondary school and 17.8% were planning to find a job. The mean age of planning to get married was 25.6 (SD = 4.6) years old for girls (n = 93) and 26.9 (SD = 4.8) years old for boys (n = 114).

**Table 1:** Demographics of Subjects

	Girls		Boys	
	n = 153	(%)	n = 152	(%)
Age				
11 to 12	76	(49.7)	48	(31.6)
13	60	(39.2)	48	(31.6)
14 to 16	17	(11.1)	56	(36.8)
Grade				
6th grade	89	(58.2)	80	(52.6)
7th grade	64	(41.8)	72	(47.4)
Religion				
Christian	70	(45.8)	71	(46.7)
Muslim	83	(54.2)	81	(53.3)
Future plan				
Go to secondary school	130	(85.0)	109	(71.7)
Find a job	19	(12.4)	27	(17.8)
Do not know	4	(2.6)	15	(9.9)
Stay home	0		1	(0.7)
Number of brothers and sisters				
mean [SD]	2.2	[2.0]	2.7	[2.4]
Age for planning to marry				
mean [SD]*	15.6	[13.0]	20.1	[12.4]

*Girls n = 93, Boys n = 114

Sexual experience in girls and boys differed significantly (χ^2^*(1) *= 7.282, p = 0.007). Approximately 27% of the girls had sexual experience and among the girls who had sexual experience, 51.2% reported that it was by force (Table 2). For the boys, 41.4% reported having had experience of sex and among the boys who had sexual experience, 36.5% reported that it was by force.

**Table 2:** Percentage of girls and boys who have experience of sex

	Girls		Boys		chi-square	p-value
	n = 153	(%)	n = 152	(%)		
Experience of sex						
Not yet	112	(73.2)	89	(58.6)	7.282	0.007
Yes	41	(26.8)	63	(41.4)		

By force	21	(51.2)	23	(36.5)	-	
Willingly	20	(48.8)	40	(63.5)		

Communication with the students and their parents differed between girls and boys. More girls than boys communicated with their parents about their daily life; 73.2% of the girls communicated with their parents, while for boys it was 65.1%. Concerning communication about sex and HIV/AIDS, it was 37.3% for the girls and 29.6% for the boys.

### Scores in knowledge, attitude, and behavior tests in relationship to communication

Table 3 describes the comparison in knowledge, attitude and behavior tests in relationship to girls' communication with parents. When dividing the group according to communication with parents about daily life, the "Yes" group's mean score was 6.0 and the "No" group's was 5.5, which showed significantly higher scores in the knowledge test (*t *= 2.0, *p *= 0.05). In the attitude test, the "Yes" group's mean score was 31.1 and 29.0 in the "No" group, which showed significantly higher scores (*t *= 2.7, *p *= 0.007). There was no statistically significant difference in behavior test scores.

**Table 3:** Comparison of communication with parents by knowledge, attitude, behavior score in pre-test (Girls n = 153)

	n	score	SD	t-value	p-value
Daily life					
Knowledge					
Yes	112	6.0	[1.4]	2.0	0.05
No	41	5.5	[1.5]		
Attitude					
Yes	112	31.1	[4.4]	2.7	0.007
No	41	29.0	[4.1]		
Behavior					
Yes	112	26.1	[3.4]	1.6	0.102
No	41	25.0	[4.1]		

Sex and HIV/AIDS					
Knowledge					
Yes	57	6.2	[1.4]	1.9	0.062
No	96	5.7	[1.4]		
Attitude					
Yes	57	31.6	[3.8]	2.9	0.018
No	96	30.0	[4.7]		
Behavior					
Yes	57	26.4	[3.1]	1.5	0.141
No	96	25.5	[3.9]		

When dividing the group according to communication with parents about HIV/AIDS, the "Yes" group's mean score was 31.6 and the "No" group's was 30.0, which showed significantly higher scores in attitude tests (*t *= 2.9, *p *= 0.018). There were no statistically significant differences in knowledge and behavior test scores. Girls who communicated with their parents had higher attitude scores compared to those who did not communicate with their parents.

Table 4 describes the boys' scores. When dividing the group according to communication with parents about daily life and about sex and HIV/AIDS, neither of the tests showed a statistical difference.

**Table 4:** Comparison of communication with parents by knowledge, attitude, behavior score in pre-test (Boys n = 152)

	n	score	SD	t-value	p-value
Daily life					
Knowledge					
Yes	99	6.5	[1.5]	1.2	0.248
No	53	6.2	[1.2]		
Attitude					
Yes	99	31.2	[3.6]	1.7	0.097
No	53	30.0	[4.5]		
Behavior					
Yes	99	25.5	[4.1]	0.2	0.826
No	53	25.7	[3.5]		

Sex and HIV/AIDS					
Knowledge					
Yes	45	6.5	[1.4]	0.6	0.568
No	107	6.4	[1.4]		
Attitude					
Yes	45	31.3	[3.9]	1.0	0.341
No	107	30.6	[4.0]		
Behavior					
Yes	45	26.2	[3.5]	1.4	0.176
No	107	25.3	[4.0]		

"Communication with parents about daily life" and "HIV/AIDS" influenced girls' attitude score. The knowledge score was higher in girls who communicated with their parents about daily life than those who did not communicate with their parents. However, "communication with their parents" did not show differences in the behavior scores. In addition, no statistically significant difference was shown in either of the tests for the boys.

### Comparison before and after the program

Table 5 describes the scores of knowledge, attitude, and behavior before and after the program. The scores in the knowledge test and the behavior test increased after the program for both girls and boys. The girls' mean score in the knowledge pre-test was 5.9 and 6.8 in the post-test, which was a significant increase (*t *= 7.9, *p *= 0.000). The mean behavior pre-test score was 25.8, and 26.6 in the post-test, which showed a significant increase (*t *= 3.0, *p *= 0.003). However, the attitude score did not show a statistically significant difference between pre-test and post-test.

**Table 5:** Comparison of pre-test and post-test values of girls and boys

	Girls n = 153						Boys n = 152					
	Pre-test		Post-test		t-value	p-value	Pre-test		Post-test		t-value	p-value
Knowledge												
mean [SD]	5.9	[1.4]	6.8	[1.0]	7.9	0.000	6.4	[1.4]	7.0	[0.8]	4.5	0.000
Attitude												
mean [SD]	30.5	[4.4]	30.7	[4.4]	0.4	0.666	30.8	[3.9]	30.8	[4.2]	0.0	0.973
Behavior												
mean [SD]	25.8	[3.6]	26.6	[3.4]	3.0	0.003	25.6	[3.9]	25.6	[3.9]	2.4	0.019

The boys' mean score in the knowledge pre-test was 6.4 and 7.0 in post-test, which increased significantly (*t *= 4.5, *p *= 0.000). The mean behavior pre-test score was 25.6 and 26.4 in the post-test, which showed a significant increase (*t *= 2.4, *p *= 0.019). However, the attitude score did not show a statistically significant difference between pre-test and post-test.

### Scores in pre-test

The girls' highest score in pre-test was 92.2%, which was "protecting themselves from HIV/AIDS". The next was "girls' maturity signs (88.9%)" and "jumping prevents pregnancy (83.0%)". The lowest score was "monthly vaginal blood (50.3%)", followed by "difficulty of getting HIV/AIDS (64.7%)" and "girls at puberty ovulate (65.4%)".

The boys' highest score was "protecting themselves from HIV/AIDS (98.0%)". Next was "girls' maturity signs (91.4%)" and "jumping prevents pregnancy (82.9%)". The lowest score was "difficulty of getting HIV/AIDS (30.9%)", followed by "girls at puberty ovulate (50.7%)", "monthly vaginal blood (67.8%)" and "boys' maturity signs (67.8%)".

The highest percentage of correct scores in both girls and boys was the same, which was: "protecting themselves from HIV/AIDS", "girls' maturity signs" and "jumping prevents pregnancy". The lowest percentage of correct scores was the same in both groups: "monthly vaginal blood", "difficulty of getting HIV/AIDS" and "girls at puberty ovulate" (Table 6).

**Table 6:** Knowledge of reproductive health test items by percentage of correct answers by girls and boys

		Percentage of correct answer			
		**Girls (n = 153)**		**Boys (n = 152)**	
	**Statement**	**Pre-test**	**Post-test**	**Pre-test**	**Post-test**

1	Girls at puberty, ovulates every month.(True)	65.4	90.8	50.7	92.1
2	Girls mature signs: breast develops.(True)	88.9	95.4	91.4	96.7
3	Boys mature signs: voice deepens.(True)	71.9	96.7	67.8	95.4
4	Puberty girls will not become pregnant.(False)	80.4	94.8	78.9	86.8
5	Jumping and washing prevents pregnancy.(False)	83.0	96.1	82.9	94.7
6	Puberty boys can impregnate girls.(True)	75.8	96.7	80.9	98.0
7	Monthly vaginal blood is normal for puberty girls.(True)	50.3	83.7	67.8	93.4
8	Condoms should be given to avoid pregnancy and diseases.(True)	73.9	79.7	75.0	85.5
9	Everyone can protect themselves from HIV/AIDS.(True)	92.2	98.7	98.0	96.7
10	Difficult to get HIV/AIDS.(False)	64.7	69.9	30.9	78.3

### Scores in post-test

For the girls, the percentage of the correct answers increased. Seven out of ten items were over 90% (Table 6). In the post-test almost all of the girls chose the correct answer about "protecting themselves from HIV/AIDS (98.7%)", and a similar increase was seen in "boys' maturity signs (96.7%)" and "boys can impregnate girls (96.7%)". However, items such as "difficulty of getting HIV/AIDS (69.9%)", "condom use to avoid pregnancy and diseases (79.7%)" was less than 80%.

For the boys, the percentage of the correct answers increased also. Seven out of ten items were over 90%. In the post-test almost all of the boys chose the correct answer about "boys impregnating girls (98.0%)", "girls' maturity signs (96.7%)", and "protecting themselves from HIV/AIDS (96.7%)". However, the item "difficulty of getting HIV/AIDS (78.3%)" was less than 80%. Therefore, there was not a big difference in both girls and boys in the item "difficulty of getting HIV/AIDS".

### Process evaluation

The time for process evaluation was approximately 20 minutes. The participants were volunteers who remained after class. The participants reported that the time for discussion was too short and they wanted more time. They gained new knowledge about how they can escape from temptation and select good friends. Most of them found it challenging to communicate with their parents frequently as a good way to express their feelings and problems. The picture drama used in the program was closely related to their school life situation; therefore, it touched their feelings. About the venue, the desks and chairs were not enough for all the students to sit comfortably. Furthermore, they proposed to have more frequent reproductive health education including sex education like other studies they have at school.

## Discussion

### Efficacy of reproductive education program

The objective of this study was to evaluate a reproductive health awareness program for the improvement of reproductive health for unmarried adolescents using a questionnaire assessing their knowledge, attitude, and behavior.

The findings indicated an increase in knowledge and behavior that showed a statistically significant difference between pre-test and post-test for both girls and boys. However, the attitude score did not show a statistically significant difference between pre-test and post-test.

Likewise, a randomized trial with a pre-test and post-test research design showed an increase in knowledge but no statistically significant difference in attitude between groups at pre-test and post-test, in their evaluation of an AIDS education program designed for young adults [22]. Another study conducted about abstinence-based small group pregnancy prevention showed no short-term differences between groups in attitude towards teenage pregnancy[23]. In addition, the study by Gallant and Maticka-Tydale found that knowledge and attitudes are easy to change, while changing behavior is challenging [16]. Similar results appeared in the systematic review by Paul-Ebohimhen et al., which reviewed 23 articles and reported that knowledge and attitude were most likely to change, while behavior changes were less likely to occur [24].

Therefore school-based programs were effective for knowledge improvement, but attitude may be difficult to change. This program seems to be more accessible to the students, using picture drama with apron material and small group discussion. This program is a feasible program for other areas in Tanzania.

### Program evaluation

Gallant and Maticka-Tydale compared reproductive health education programs applying the following criteria: theory; school level; number of schools included; community involved or not; content, which includes targeted behavior and main activities; form, which includes in/after school and total exposure; and implementation, which includes instructors, instructor training, and monitoring [16].

The strengths of our research is that (1) we targeted young school adolescents aged 11 to 16, (2) we conducted the education program for 305 students which included both girls and boys, (3) we targeted abstinence and condoms, (4) we used materials that were easy to understand for young students. The weaknesses are: (1) The program was only 45 minutes, and students said that they wanted more time for discussion, (2) because the program evaluation was conducted immediately after the program, we cannot exclude the possibility of retention.

### Communication with parents

The results showed that the adolescent girls who communicate more with their parents had significantly higher knowledge and attitude about reproductive health than those who did not communicate. Yet another report of parents considered sexual communication difficult and embarrassing [25]. Additionally, parents attempted to communicate with their children, although it was difficult when there was lack of knowledge [26]. Wamoyi et al. explored parent-child communication about sexual and reproductive health in families through participant observation, in-depth interviews, and focus group discussions in rural Tanzania [26]. They reported that communication about HIV/AIDS and sexually transmitted infections were commonly discussed in families. In addition, the feeling of parent-child closeness was very important in determining the parent-child relationship and communication about sexual and reproductive health. Among adolescents, ages 13 to 17 had no communication with their parents about sexual topics before they started to engage in sexual intercourse [27]. From the results of this study and others, we found that if there is sufficient communication about daily life and sexual topics between parents and adolescents the teenagers may be able to change their attitudes.

Furthermore, using a randomized controlled trial evaluation of parents talking with their adolescent, it was found that work-place programs can have a positive effect between parents and their adolescent to improve sexual health communication [28]. The intervention contents *talking parents, healthy teens*, provided eight weekly one-hour sessions with a group of about 15 parents with children in grades 6 to 10 at their work site during the lunch hour. They reported those adolescents whose parents talked to them about sexuality were more likely to delay intercourse, use contraception and have fewer sexual partners. Among the total of 312 adolescents, it was found that repetition of sexual communication between parents and their adolescent was an important predictor of the teenager's perceptions [29]. These findings had a similar direction as the results of this study and supported the development of our program for parents.

### Environment surrounding adolescents

In this study 26.8% of the girls and 41.4% of the boys already had sexual experience. This proportion is similar to the study conducted in Tanzania [10] about sexual practice of adolescents that reported that 21.1% of girls and 42.6% of boys had sexual experience. Also, another report indicated that girls who had sexual experience comprise 20.9%, and boys who had sexual experience comprised 51.2% [18].

In this survey what was not so obvious or easily reported was that 51.2% of the girls reported that their experience of sex was by force. Another study reported the dating violence among school students in Tanzania; 37.8% of the females had been victims and 21.8% of males were perpetrators. The number of victims of sexual violence is considered a reliable number for Dar er Salaam in Tanzania [30]. According to the results that adolescents are in danger of forced sex or early debut of having sex, it is important for them to have the correct knowledge and the decision-making ability that our study focused on.

### Limitations of the study

There are several limitations of this study that should be noted. First, the results may not be generalizable to all school adolescents in urban Tanzania as only three schools were selected. There is a need to expand the participants to students in rural Tanzania. Second, since this study evaluated the outcomes immediately after the program, it is not certain what the knowledge retention is and for how long it will be retained. In the future, we will need to evaluate the program's effectiveness over the long-term. Third, the adolescents may not have given accurate answers to questions about sexual activities; they may have over or under reported their behavior. Lastly, the instrument used in this study is in its early use. Therefore, we will need to consider the validity and reliability from accumulating data.

## Conclusions

Teenagers have sexual experiences including sexual violence. Both of these phenomena are prevalent among school-going adolescents before they have had appropriate knowledge about reproductive health, thereby putting them at great risk.

The reproductive health program improved the students' knowledge and behavior about sexuality and decision-making after the program for both girls and boys. However, their attitudes about reproductive health were not likely to change based on the educational intervention as designed for this study.

## Competing interests

The authors declare that they have no competing interests.

## Authors' contributions

FM collected data and FM and MI analyzed data. FM and SH and MI participated in reviewing and drafting the manuscript in all stages. All authors read and approved the final manuscript.
